# Obesity: An Underlying Risk for Acute Aortic Dissection

**DOI:** 10.3390/jcm14217876

**Published:** 2025-11-06

**Authors:** Han Zhang, Yu Lun, Jian Zhang

**Affiliations:** Department of Vascular Surgery, The First Hospital of China Medical University, Nanjing Bei St. 155, Shenyang 110001, China

**Keywords:** aortic dissection, age, obesity, metabolism, vascular

## Abstract

Obesity is a significant risk factor for cardiovascular diseases. Although previous studies have shown uncertainty about its role in aortic dissection (AD), our clinical observations showed that most younger patients with acute AD have a significantly higher body mass index. The underlying reasons are yet to be investigated. Recent studies have suggested that obesity is linked to vascular pathophysiology, including endothelial injury, medial remodeling and deficiency, perivascular adipose tissue dysfunction, and systemic dysfunction. Understanding the association between obesity and acute AD can aid in recognizing high-risk populations, providing an earlier chance of diagnosis and intervention, and improving clinical outcomes for acute AD in young obese patients. This review analyzes and integrates current data to explain the potential role of obesity in acute AD pathogenesis.

## 1. Introduction

Acute aortic dissection (AD) is a life-threatening vascular disease where blood penetrates the aortic media through a tear in the aortic intima, forming perfused channels that expand along the long axis of the aorta, creating a new lumen within the aortic wall. The incidence of acute AD is increasing in China, resulting in high mortality and morbidity rates [1]. The precise mechanism is still unknown, but factors such as inflammatory infiltration, immune response, oxidative stress, local lesions, and abnormal matrix metabolism are implicated in the pathogenesis [2]. Our clinical observations suggest that young obese men are more prone to acute AD, and our and other recent studies have confirmed that younger patients with acute AD have a higher body mass index (BMI) than middle-aged and older patients [3,4]. However, this remains an observational association. The absence of adjusted analyses for confounders means this signal still warrants further investigation.

Obesity is a significant risk factor for metabolic and cardiovascular diseases (CVDs), with significant evidence linking it to increased morbidity and mortality rates. Adipose tissue (AT) is a dynamic organ with the potential to expand unlimitedly during obesity and is distributed throughout the body in two primary types: White adipose tissue (WAT) and Brown adipose tissue (BAT). WAT composes the largest proportion of the body’s AT and is distributed particularly around major organs and blood vessels in the abdomen and subcutaneously. Excessive accumulation of WAT, particularly in the visceral area, increases the risk of cardiac metabolic abnormality, hypertension, and CVD [5]. In contrast, BAT composes only 4.3% of all AT in the body and is found in cervical paraspinal, mediastinal, and axillary regions. BAT, which accounts for most of the perivascular adipose tissue (PVAT) surrounding the thoracic aorta, is resistant to obesity-induced inflammation and takes on a protective role [6]. Recent clinical and experimental studies suggest that AT serves as an endocrine organ with immune functions in addition to regulating energy homeostasis [7,8,9]. These findings suggest a possible link between obesity and acute AD occurrence in young patients with obesity. This review focuses on recent research regarding the effects of obesity on the aortic wall and discusses causality and mechanisms involved in acute AD occurrence and development.

## 2. Epidemiology of Obesity and Acute Aortic Dissection

Previous studies have reported that acute AD commonly occurs in older adults, with an average age of 63.1 years in Europe and the United States, with a higher incidence rate in winter and spring and the lowest in summer [10]. Population-based studies in the United States have estimated the incidence of AD to be between 2.6 and 3.5 cases per 100,000 person-years [11,12]. However, a 10-year study from 2002 to 2012 in the United Kingdom reported a higher incidence of 6 per 100,000 person-years, including out-of-hospital deaths [13]. According to the 2010 Office for National Statistics population projections, the number of dissection events in the UK is expected to increase from 3892 in 2010 to 6893 in 2050 for men and women, with the majority occurring in those aged > 75 years [13,14]. In data from the Swedish National Patient Register and the Cause of Death Register, the incidence of acute AD in the general population has reached 7.2 per 100,000 person-years [15]. These studies suggest that the incidence of acute AD is increasing in Western countries, potentially due to increased clinical awareness of the disease and the availability and use of imaging techniques, particularly Computed Tomography (CT), in emergency departments [16].

Currently, there is a lack of population-based epidemiological data on acute AD in China. However, a study based on 2011 China Health Insurance Research data found that the annual incidence of AD in China was 2.8 per 100,000, with a higher prevalence in northern China than in southern China [17,18]. The significantly lower incidence of acute AD compared to Western countries may be due to the underdiagnosis of some patients at the time of pre-hospital death and limitations in data coverage by the insurance industry. Patients with acute AD tend to be younger in northern China than in southern China, with an average age of onset at 52.7 years, which is approximately 10 years lower than that in Europe and the United States [19,20,21]. In addition, the onset of AD in China follows a seasonal pattern, with a higher incidence in winter compared to summer [22], particularly in northern China, where winters are longer and colder.

Obesity and its associated diseases were previously thought to be mainly prevalent in Western countries. However, since 1980, many countries, including China, have shown a continuous increase in the prevalence of obesity and average BMI due to changes in diet and lifestyle, making China the country with the highest overweight or obese population in the world [23,24,25]. While the relationship between obesity and acute AD remains controversial despite evidence of a strong association between obesity and various vascular diseases, a quantitative synthesis of available evidence can strengthen this link. Although direct, large-scale meta-analyses on BMI and AD incidence are limited, a preliminary analysis by The International Registry of Acute Aortic Dissection (IRAD) showed that patients with acute AD with a higher BMI are younger—for every 5-unit increase in BMI, the age of AD onset decreased by 3 to 5 years—and more likely to have hypertension and/or diabetes [6,26,27]. This aligns with findings from Chinese cohorts. Our previous study, along with others from southern China, found that the age of patients with acute AD in China tends to be younger, with higher BMI and body surface area [3,28]. This is particularly relevant given the significantly higher prevalence of obesity in northern China [29,30], which parallels the region’s higher AD incidence and younger patient age.

While the epidemiological data highlight a clear association between obesity, younger onset age, and AD, the inherent limitations of these primarily observational studies must be acknowledged. These associations can be influenced by unmeasured confounding factors, such as genetic predispositions, specific dietary patterns, or other comorbidities not included in the registry data. As this is a narrative review without a primary dataset for analysis, we are unable to perform adjusted analyses to control for potential confounders. Furthermore, regional variations in incidence rates may reflect differences in diagnostic capabilities and healthcare access rather than true biological differences. Therefore, the current epidemiological evidence establishes a correlation between obesity and aortic dissection, but does not prove causality. Although obesity is one of the risk factors for hypertension and is often considered an indirect factor in acute AD development, obesity-induced systemic inflammatory states and altered PVAT function may have a direct impact on the occurrence and progression of acute AD. Future studies with prospective designs are urgently needed to quantify the risk of aortic dissection in obesity populations and elucidate the underlying mechanisms.

## 3. The Role of PVAT in Aortic Physiology and Pathophysiology

While AT functions similarly in animal models and humans, its location and abundance can vary greatly. PVAT, which composes the outer matrix surrounding aorta and systemic blood vessels, contributes to vascular physiology and pathophysiology in an “outside-in” pattern [31]. The predominant cell type in PVAT is adipocytes, but it also contains stromal components, such as preadipocytes, stem cells, and fibroblasts; inflammatory cells, such as macrophages, lymphocytes, and eosinophils; and nerve and vascular cells, forming the AT microvascular network that surrounds most of the blood vessels except cerebral vessels [32,33]. PVAT has a lower degree of differentiation compared to classical visceral AT and is more similar to preadipocytes [34]. The phenotype of thoracic PVAT is similar to BAT, while abdominal PVAT is more like WAT [35]. Beige adipose tissue (BeAT), a type of inducible brown AT, is also present in mice and humans and tends to be scattered in WAT, but it also expresses uncoupling protein-1 as brown adipocytes [5]. In addition, BeAT can convert into dual directions, such as BAT or WAT, during adipose remodeling. PVAT releases adipokines, cytokines, and chemokines in addition to providing mechanical support for aorta [36]. The paracrine/endocrine nature of PVAT allows these factors to impact intravascular homeostasis and function by reaching the endothelium through the outer and middle layers of the vasculature [37,38].

PVAT inhibits inflammatory processes by releasing anti-inflammatory adipokines, such as lipocalin, reticulin, Fibroblast Growth Factor 21 (FGF21), and nitric oxide (NO), which improve free fatty acid metabolism and promote vasodilation [38]. However, in cases of obesity, PVAT volume increases and becomes dysfunctional, contributing to macrophage infiltration and changes in cell composition, molecular characteristics, and large-scale secretion of pro-inflammatory and anti-vasodilatory factors, such as leptin, resistin, Tumor Necrosis Factor-Alpha (TNF-α), monocyte chemoattractant peptide-1 (MCP-1), Interleukin 6 (IL-6), and Interleukin 1(IL-1) [39]. This infiltration and local oxidative stress trigger an “outside-in” signal in the aortic wall, leading to vascular endothelial and smooth muscle cell dysfunction, which can result in pathological conditions [40,41].

## 4. Abnormal Endothelial Function in Obesity Triggers Acute AD

The pathogenesis of vascular remodeling is associated with various vascular diseases and is initiated by impaired endothelial function and recruitment of peripheral circulating inflammatory cells to the intima and medial layer, as seen in acute AD occurrence and development [2,42]. An intact endothelial function has been shown to prevent AD formation. A recent study found that high levels of inducible nitric oxide synthase (iNOS) can promote excessive production of NO, which may contribute to AD development [43]. A study by Pan et al. identified the molecular mechanisms underlying AD formation, demonstrating that NO overproduction and subsequent S-nitrosylation of plastin-3, an actin-binding protein, induce endothelial barrier dysfunction and cause AD [44]. In addition, iNOS inhibition reduces the effect of S-nitrosylation of plastin-3 and regulates endothelial barrier function [44].

Constant exposure of the endothelial layer to hyperlipidemia in obesity results in the internalization of lipids, which contributes to endothelial activation and progressive accumulation of lipids within the intima. This accumulation of lipids enhances endothelial permeability by increasing the expression of cytokines, chemokines, and adhesion molecules [45]. Endothelial permeability is partly regulated by the dynamic opening and closure of cell–cell adherens junctions. Vascular endothelial cadherin (VEC) is the most important transmembrane component of endothelial adherens junctions and is exclusively expressed by endothelial cells. It is vital for the formation and integrity of blood vessels. Increased levels of soluble VEC and auto-antibodies to human vascular endothelial–cadherin suggest endothelial dysfunction in humans [46]. The disruption of inter-endothelial junctions among endothelial cells occurs in the early stages of endothelial barrier dysfunction [47], which is mostly the underlying risk of acute AD in patients with obesity. Our recent study shows a significant increase in soluble VEC in the peripheral blood of patients with AD, indicating impaired endothelial cell–cell adherens junctions and endothelial dysfunction in the initial stage of acute AD [48].

Obesity leads to increased adipocyte volume and whitening of PVAT in the thoracic aorta [49], which impairs both the mechanical protection of the aorta [33] and endocrine and paracrine actions [50]. In addition to releasing adipokines, whitening PVAT produces classical chemokines, including IL-6, IL-8, and MCP-1 [51], and becomes more inflammatory by attracting macrophages and T lymphocytes into PVAT. In obesity, macrophages within WAT exhibited increased proliferation. This is associated with a significant increase in MCP-1 expression, and MCP-1 mRNA levels in AT samples correlate with the degree of obesity [52]. Adipocyte-produced MCP-1 contributes to macrophage infiltration into AT and local proliferation, increasing AT inflammation [53]. TNF-α, expressed by macrophages, has pro-inflammatory effects, and can promote the production of other pro-inflammatory factors, such as IL-6, leptin, and resistin [54]. Excessive expression of MCP-1 and TNF-α in transplanted PVATs can exacerbate endothelial dysfunction in distal vessels by enhancing the inflammatory response [55]. PVAT expresses a complex reactive oxygen species (ROS)/reactive nitrogen species, including NADPH Oxidase 2 (NOX2), endothelial nitric oxide synthase (eNOS), and superoxide dismutase (SOD) isoforms [56]. Although the appearance of PVAT in the obese aorta may not suggest a change in endothelium-dependent vasodilation, a study has shown that PVAT promotes endothelial dysfunction through increased NOX2-derived oxidative stress and production of pro-inflammatory cytokines in cases of diet-induced obesity [57]. These changes in PVAT are associated with the mechanical weakening of the endothelial barrier [33], compromising the integrity of the intimal structure and becoming a precondition for patients with obesity with acute AD.

## 5. PVAT Dysfunction-Induced Aortic Remodeling in Obesity

Adipocytes in the PVAT of the aortic arch area, where acute AD primarily begins and the predominant tear occurs, belong to a distinct population that develops differently from other periaortic AT [58]. They originate from smooth muscle protein 22-Alpha (SM22α^+^) progenitors and neural crest cells, which share the same progenitors as smooth muscle cells rather than Myf5^+^ progenitors [49]. PVAT is a unique type of AT that not only mechanically supports the aortic vasculature but also plays a crucial role in vascular homeostasis. However, in obesity, PVAT undergoes a transformation from BAT to WAT [49,59] and contributes to vascular remodeling through the infiltration of pro-inflammatory macrophages and T lymphocytes, as described earlier. In acute AD, inflammatory cells such as T lymphocytes and macrophages have been observed diffusely throughout the media or focal accumulations between the smooth muscle cell (SMC) layer and inside the vasa vasorum wall, indicating their possible immigration from adventitia to the media of the aortic wall [60]. The proximity of PVAT to adventitia facilitates the transfer and spread of local inflammation induced by dysfunctional PVAT to the aortic wall via paracrine secretion. Therefore, in obesity, PVAT may initiate a series of responses presumed to be the primary mechanism by which PVAT contributes to acute AD pathogenesis [59]. The inflammatory cells are a potential source of proteases that can degrade the extracellular matrix (ECM) and weaken the aortic wall. Matrix metalloproteinase-9 (MMP-9), matrix metalloproteinase-2 (MMP-2), elastase, and collagenase secreted from macrophages can degrade the ECM and intercellular junctions, leading to detachment of SMCs from the ECM and cell death and leaving the aorta more vulnerable to dissection [61]. Previous studies identified increased MMP-2 and MMP-9 expressions at the site of the intimal tear, at the surface of disrupted elastic fibers, and at the border of the areas of medial degeneration in AD [62], indicating that ECM destruction by MMPs and aortic wall remodeling may occur before acute AD onset.

PVAT-derived adipokines, cytokines, ROS, and gaseous compounds contribute to vascular remodeling. Adiponectin is one of the most abundant adipokines generated by PVAT and modulates immune cell activity in AT, including macrophages, eosinophils, and mast cells. It also forms different molecular weights of the complex, while the high-molecular-weight complex blocks Nuclear Factor Kappa B (NF-κB) activation and inhibits pro-inflammatory cytokine production [6]. However, adiponectin serum levels decrease in obesity [63] due to adiponectin transcription and translation suppression in an adipocyte cell line by TNF, IL-6, and other pro-inflammatory mediators [64]. Leptin, another abundant adipokine in AT, enhances pro-inflammatory cytokine production in peripheral blood monocytes and macrophages and induces ROS production in macrophages, neutrophils, and endothelial cells [24]. Leptin primarily acts on leptin receptors in the endothelium to mediate vasodilation through an NO-dependent and independent mechanism [65]. In addition, leptin regulates their functions by affecting leukocyte chemotaxis, oxygen radical release, vascular SMC proliferation, and adhesion molecule expression on endothelial cells and VSMCs [66]. Resistin, mainly found in WAT and expressed in monocytes and macrophages, induces pro-inflammatory effects through NF-κB signaling activation by its receptor cyclase associated actin cytoskeleton regulatory protein 1 (CAP-1). Through this activation, resistin binds to toll like receptor 4 (TLR-4) and regulates TNF-α and IL-6 production in macrophages [67]. It also induces endothelial dysfunction by upregulating vascular cell adhesion molecule 1 (VCAM-1) and intercellular adhesion molecule 1 (ICAM1) expressions and inducing C-C motif chemokine ligand 2 (CCL2) and endothelin-1 from endothelial cells [36]. PVAT secretes numerous other adipokines, and in obesity, their altered secretions intensify inflammation and oxidative stress, contributing to aortic wall remodeling [68].

Obesity also increases the risk of acute AD by increasing aortic stiffness through PVAT. The quality and quantity of ECM, including elastin and collagen, largely determine aortic stiffness. They contribute to vascular elasticity and compliance as well as strength and stiffness. An abnormal quantity and quality of ECM, such as decreased elastin synthesis and increased collagen content [69], as seen in obesity, are closely associated with the change in aortic stiffness. In obese mice, elastic fiber fragmentation significantly increases, impairing the cushioning effect of the aorta and contributing to increased aortic stiffness [70]. Aortic stiffness in obesity is associated with structural and functional changes in the intima, media, and adventitia. These changes include impaired endothelial NO release, increased oxidative stress and endothelial permeability, and stimulation of the inflammatory micro-environment, all of which significantly influence aortic function and stiffness. This leads to constant vascular constriction and enhanced tissue remodeling, including fibrosis, making the aortic wall vulnerable to dissection.

Although these mechanistic studies provide compelling evidence for the role of endothelial dysfunction and MMP activity in AD, most animal models of AD involve gene editing or chemical induction and may not fully simulate the chronic, low-grade inflammatory state features of obesity-associated AD in humans.

## 6. Obesity-Induced Systemic Dysfunction Is Linked to Aortic Injury

In obesity, increased basal lipolysis is associated with excess fatty acid production, impairing systemic insulin sensitivity and promoting AT inflammation. Ectopic accumulation of circulating fatty acids in insulin-responsive tissues can further exacerbate insulin resistance, reducing the normal insulin response [71]. In normal physiological conditions, insulin regulates vascular tone and maintains vascular endothelial cell function by regulating eNOS gene expression to produce NO. Pathologically, activation of protein kinase C in vascular tissue during insulin resistance can inhibit phosphatidylinositol-3-kinase and eNOS activities, leading to endothelial dysfunction [72].

Obesity is a significant risk factor for hypertension and can increase blood pressure via several mechanisms, including overactive renin-angiotensin system [73], increased arterial stiffness, and PVAT dysfunction. PVAT with changes from BAT to WAT loses its anti-contractile effect and contributes to hypertension [31]. In turn, hypertension can impair endothelial cell junctions and contribute to dysfunctional endothelial cell junctions, decreasing spatial control of endothelial Ca^2+^ signaling and reducing the expression of endothelial tight junction proteins [74]. These changes increase the risk of acute AD. Hypertension can also induce a hypercontractile state and dedifferentiation of vascular smooth muscle cells to a proliferative or migratory phenotype, contributing to aortic remodeling and increasing the risk of acute AD [75].

Obesity is associated with excessive AT, inducing hypertrophy and hyperplasia of adipocytes. However, capillary density and function in AT are inadequate, resulting in lower capillary density, concomitant insulin resistance, impaired AT capillaries, and higher AT inflammation [76]. These factors are collectively linked to a local hypoxic response in obese AT. AT hypoxia stabilizes and activates hepatic ischemic factor (HIF), a key transcription factor that mediates hypoxia-induced adipocyte dysfunction and inflammatory response, contributing to obesity-related insulin resistance and macrophage accumulation [77]. Hypoxia and excessive free fatty acids released from obese AT promote macrophage-mediated inflammatory responses [78], and the increased ROS in the hypoxic AT not only propagates hypoxic signals but also activates and stabilizes HIF-1α and induces a large amount of pro-inflammatory factors production, such as TNF-α, IL-1β, and IL-6 [79]. These pro-inflammatory factors infiltrate the bloodstream, contributing to chronic and systemic inflammatory states. When endothelial cells are exposed to these inflammatory factors, they can not only produce adhesion factors to provide attachment conditions for immune cells, but also secrete chemokines to attract and activate inflammatory cells such as macrophages and lymphocytes, causing them to gather in the aorta [80]. Upon infiltrating immune cells in the medial layer of the aorta, it releases inflammatory factors and matrix metalloproteinases, which create a self-sustaining inflammatory stimulus. This process not only promotes the degradation of the extracellular matrix but also suppresses the synthesis of new matrix components, ultimately contributing to medial degeneration [81,82]. Similarly, driven by chronic inflammation originating from adipose tissue, the adventitial layer also undergoes progressive fibrosis. Under the combined action of these factors, the aortic wall structure becomes fragile, laying the foundation for aortic dissection formation.

Current evidence suggests that the above mechanisms are not independent of each other, but constitute a vicious circle of synergistic interactions that jointly promote the occurrence of aortic dissection. As an accelerator of disease, insulin resistance not only directly causes or exacerbates high blood pressure, but is also associated with dysfunction of PVAT, causing it to release more pro-inflammatory factors and reactive oxygen species [76]. Dysfunctional PVAT delivers its inflammation directly to the aortic wall, contributing to vascular inflammation, oxidative stress, and matrix degradation [83]. This local inflammatory amplification effect makes the aortic wall more fragile and more sensitive to the damaging effects of hypertension. Then, hypertension provides the ultimate mechanical force, transforming structural fragility into an acute AD event.

## 7. Causality and Mechanism of Acute AD in Obesity

Acute AD occurs when blood flow suddenly enters the aortic media through a spontaneous intimal tear in a state of severe hypertension, causing abrupt and serious back and chest pain. Estimating the prior risk of aortic dissection by assessing predisposing conditions is recommended by the American College of Cardiology, the American Heart Association, and The European Society of Cardiology [84,85,86]. We believe that obesity-induced aorta-specific alterations and systemic dysfunction are the underlying reasons for acute AD pathogenesis. Obesity contributes to low-grade inflammation that can progress to hypertension and acute AD. The most frequent site of primary tear is at the aortic arch, where the altered direction of the blood flow due to curvature causes increased shear stress and tremendous strain on the aortic wall at the isthmus. Obesity-induced endothelial dysfunction and disrupted inter-endothelial junctions make the aorta vulnerable to this sudden stress, especially in severe hypertension. Strong blood flow can tear the intima and rush into the aortic media that has already been destructed and remodeled in obesity. This flow forms a false lumen in the aortic wall is linked to acute AD.

Obesity can alter the wall shear stress profile of the aorta independently of hypertension. Obesity is accompanied by hyperlipidemia and increased cardiac output, which can contribute to elevated blood viscosity, blood flow velocity, and aortic flow, thereby directly altering the magnitude and pattern of wall shear stress [87]. The abnormal wall shear stress caused by obesity is a powerful pro-inflammatory and pro-remodeling signal that promotes chronic inflammation and structural weakening of the aortic wall, creating conditions for aortic dissection [88]. Although obesity alters shear stress, hypertension-driven circumferential wall tension is thought to be the most direct mechanical force causing initial tearing of the aortic intima [89]. When the immense wall tension generated by high blood pressure concentrates on the fragile vessel walls, it will contribute to the occurrence of aortic dissection [90]. Obesity changes wall shear stress more as a key facilitator than a major driver of intimal tearing. Hypertension provides the ultimate mechanical force required for tearing, but obesity directly weakens the structure of the vessel wall by independently altering shear stress to induce chronic inflammation of the blood vessels, greatly reducing the ability of the aortic wall to resist tearing. They together constitute a complete, lethal pathological physiological chain, contributing to the occurrence of AD.

The pathophysiological pathways linking obesity to acute AD can be understood through both indirect and direct mechanisms. A substantial portion of the risk that obesity confers for AD is likely mediated indirectly through the development of hypertension. In this pathway, obesity acts as a key driver of hypertension via mechanisms such as activation of the renin–angiotensin–aldosterone system, sympathetic nervous system overactivity, and structural vascular changes [73], with hypertension then serving as the dominant biomechanical trigger for the intimal tear. Alongside this well-established indirect route, accumulating evidence suggests that obesity may also exert potential direct effects on the aortic wall, including systemic inflammation [91] and perivascular adipose tissue dysfunction [92], which may degrade the extracellular matrix and impair endothelial function, thereby directly compromising aortic integrity, independent of its impact on blood pressure.

The relationship between obesity and acute AD is linked through many pathophysiological pathways. The most critical factors in these processes are hypertension providing mechanical force to tear the blood vessel, and matrix degradation leading to vessel wall weakness. Other factors are upstream drivers of these two factors, playing an indirect role in obesity-related acute AD. Acknowledging the complex interplay between these pathways is crucial. A limitation in definitively disentangling these direct and indirect effects lies in the nature of the available human data, which are predominantly observational and susceptible to confounding. Therefore, effectively quantifying the contribution of direct and indirect effects in the development of AD is still a problem that needs to be solved. Future studies should employ causal inference methods, constructing directed acyclic graphs to map causal hypothesis and combine it with Mendelian randomization or mediation analysis to effectively quantify the contribution of each pathway. Elucidating the relative importance between different pathways is crucial for developing targeted prevention strategies for obese individuals at risk for AD.

A large amount of evidence linking obesity to acute AD is systematically categorized in Table 1, which summarizes the results of human observational studies, animal models, and in vitro molecular studies.

## 8. Summary and Outlook

Despite various risk factors associated with acute AD, including genetics, ethnicity, hypertension, congenital factors, and geography, obesity remains a significant and essential risk. Obesity-induced intimal injury can trigger acute AD, and obesity-induced PVAT dysfunction is associated with aortic remodeling and medial deficiency, extending the initial injury to the distal aorta. Hypertension and systemic dysfunction induced by obesity further exacerbate the risk of acute AD. Therefore, it is crucial to identify high-risk patients who require intensive management. Based on the limited evidence available, clinically observed young obese men with poorly controlled blood pressure is a potentially high-risk phenotype that should be considered a positive signal for screening and prevention of AD, pending confirmation from future perspective studies that tightly control for confounding variables.

A recent study by Costa et al. confirmed a significant association between multiple anthropometric parameters and vascular disease [95], suggesting that these simple clinical indicators are not only basic background variables, but may also have important risk assessment value. Based on this, future prospective cohort studies of AD can consider integrating and analyzing specific anthropometric data with mechanistic biomarkers, so as to construct a complete chain from molecular mechanism to clinical phenotype, laying the foundation for the development of accurate risk prediction and early intervention tools. Previous studies have shown that PVAT density, as a non-invasive surrogate indicator, is strongly associated with inflammatory activity in the aortic wall [93,94,96]. Therefore, we can integrate anthropometric parameters with aortic diameter, serum inflammatory biomarkers, endothelial damage markers and PVAT density on the basis of traditional risk factors to construct a comprehensive clinical risk assessment strategy to preliminarily stratify patients. Most importantly, when patients with the aforementioned risk characteristics are present, clinicians should increase their vigilance for the prodromal symptoms of AD and consider more closer follow-up. In addition, perioperative and intensive care challenges exist in AD patients with obesity, but this is beyond the scope of this review. Elucidating how obesity affects AD postoperative recovery and long-term outcomes remains a key area of future clinical research.

This review has certain limitations that should be acknowledged. Firstly, this article is a narrative review synthesizing current knowledge on the association between obesity and acute aortic dissection, rather than systematic review of the literature. As such, the literature search was not conducted according to a pre-registered protocol, and we did not employ a PRISMA flow diagram or formal risk-of-bias assessment for the included studies. The selection of evidence was guided by the aim of providing a mechanistic and clinical overview, and thus may not encompass all existing literature on the topic. Secondly, body mass index (BMI), while useful for large-scale epidemiological studies, is a rough and non-specific indicator of obesity. Quantifying the volume, density, and inflammatory state of PVAT using readily available CT and MRI biomarkers will provide a far more direct and powerful exposure variable for understanding and predicting AD risk in obese individuals. Validating these imaging biomarkers and integrating them into clinical risk prediction models constitutes a critical and necessary next step in this field.

In conclusion, a large body of observational evidence links obesity to increased risk and early onset of AD, and mechanistic studies have elucidated plausible pathways involving PVAT dysfunction, endothelial damage, and systemic inflammation, but the direct causal relationship remains to be fully determined. Current evidence mainly includes observational studies in humans and mechanistic studies in animal models, which may not fully reflect human disease and therefore must be treated with caution regarding their impact on public health initiatives and clinical screening protocols. Future studies could use Mendelian randomization to infer causal relationships between factors, use more physiologically relevant animal models of obesity-driven AD to reveal disease pathogenesis, and conduct prospective studies to carry out standardized exposure assessments, not relying solely on BMI, but also including indicators that directly measure vascular health, such as portable blood pressure monitoring and PVAT-specific imaging biomarkers, to rigorously determine the relationship between obesity and AD. Only in this way can we reliably quantify the risk of AD in obese people and determine the clinical utility of preventing AD against obesity-related pathways.

## Figures and Tables

**Table 1:** Classification of evidence linking obesity to acute aortic dissection.

Evidence Source	Key Findings/Mechanisms Elucidated	Representative References	Evidential Strength/Limitations
Human Observational Studies			
Clinical and Epidemiological	1. Higher body mass index (BMI) correlates with younger age at AD onset. 2. Association between regional obesity prevalence and AD incidence and earlier onset age. 3. Patients with acute AD have higher BMI and body surface area.	[3,4,6,26,27,28]	Strength: Directly establishes clinical correlations and relevance in the human population. Limitation: Inherently observational; cannot prove causality and is susceptible to confounding factors.
Biomarker and Imaging	1. Elevated serum markers of endothelial injury (e.g., soluble VE-cadherin) in AD patients. 2. Reduced perivascular adipose tissue (PVAT) density on computed tomography (CT) is associated with aortic wall inflammation. 3. Increased expressions of matrix metalloproteinase (MMP)-2 and MMP-9 at the site of intimal tear in AD specimens.	[48,62,93,94]	Strength: Provides measurable, pathophysiological indicators in human patients. Limitation: Mostly cross-sectional data; difficult to establish the temporal sequence of events.
Animal Model Studies			
PVAT Dysfunction and Aortic Remodeling	1. Diet-induced obesity leads to PVAT dysfunction (transformation from brown to white phenotype, inflammation). 2. PVAT from obese animals promotes endothelial dysfunction via increased oxidative stress. 3. Obesity promotes infiltration of inflammatory cells into the aortic wall and increases expression of MMPs. 4. Elastic fiber fragmentation and increased aortic stiffness are demonstrated in obese mice.	[49,57,59,60,70]	Strength: Provides controlled in vivo evidence for mechanistic pathways and supports causality. Limitation: Physiological differences may limit direct translation to human disease.
Systemic Metabolic Dysfunction	1. Obesity models link adipose tissue hypoxia, systemic inflammation, and insulin resistance to vascular dysfunction. 2. Obesity-induced hypertension and vascular hypercontractility are demonstrated.	[73,76,78,79]	Strength: Illustrates the integrated systemic consequences of obesity on the cardiovascular system. Limitation: The complex interplay of factors can be challenging to dissect.
In Vitro/Molecular Studies			
Endothelial Dysfunction	1. Nitric oxide (NO) overproduction and S-nitrosylation of plastin-3 directly induce endothelial barrier dysfunction. 2. Adipokines (leptin, resistin) and cytokines (TNF-α, IL-6) directly impair endothelial cell function and increase adhesion molecule expression.	[44,53,54,67]	Strength: Reveals precise molecular and cellular mechanisms in a highly controlled setting. Limitation: Lacks the multicellular and hemodynamic complexity of a living organism.
Extracellular Matrix Degradation	1. MMP-2 and MMP-9 secreted from macrophages directly degrade extracellular matrix (ECM) components (elastin, collagen). 2. Pro-inflammatory cytokines suppress the synthesis of new ECM components.	[61,62,82]	Strength: Directly demonstrates the causative role of specific enzymes and factors in aortic wall weakening. Limitation: Does not recapitulate the full tissue-level biomechanical environment.

Abbreviations: AD, aortic dissection; BMI, body mass index; PVAT, perivascular adipose tissue; CT, computed tomography; NO, nitric oxide; TNF-α, Tumor Necrosis Factor-Alpha; IL-6, Interleukin-6; MMP, matrix metalloproteinase.
